# Resonance Rayleigh Scattering Spectra of an Ion-Association Complex of Naphthol Green B–Chitosan System and Its Application in the Highly Sensitive Determination of Chitosan

**DOI:** 10.3390/md14040071

**Published:** 2016-04-18

**Authors:** Weiai Zhang, Caijuan Ma, Zhengquan Su, Yan Bai

**Affiliations:** 1 School of Public Health, Guangdong Pharmaceutical University, Guangzhou 510310, China; zhangweiai0629@126.com (W.Z.); mcjane2013@163.com (C.M.); 2 Key Research Center of Liver Regulation for Hyperlipidemia SATCM/Class III Laboratory of Metabolism SATCM, Guangdong TCM Key Laboratory for Metabolic Diseases, Guangdong Pharmaceutical University, Guangzhou 510006, China

**Keywords:** chitosan, Naphthol Green B, Resonance Rayleigh scattering spectra

## Abstract

This work describes a highly-sensitive and accurate approach for the determination of chitosan (CTS) using Naphthol Green B (NGB) as a probe in the Resonance Rayleigh scattering (RRS) method. The interaction between CTS and NGB leads to notable enhancement of RRS, and the enhancement is proportional to the concentration of CTS over a certain range. Under optimum conditions, the calibration curve of Δ*I* against CTS concentration was Δ*I* = 1860.5*c* + 86.125 (*c*, µg/mL), *R^2^* = 0.9999, and the linear range and detection limit (DL) were 0.01–5.5 µg/mL and 8.87 ng/mL. Moreover, the effect of the molecular weight of CTS on the accurate quantification of CTS was studied. The experimental data were analyzed through linear regression analysis using SPSS20.0, and the molecular weight was found to have no statistical significance. This method has been applied to assay two CTS samples and obtained good recovery and reproducibility.

## 1. Introduction

Among biopolymers, chitosan (CTS), which is produced from the deacetylation of natural chitin, has seen increased use due to the presence of amino groups on the polymer backbone that make it a natural cationic polymer [1].With the extensive application of CTS in different fields [2], especially in the application of reducing weight and drug delivery system. It is very important to study the accurate and sensitive quantification of CTS for quantity monitoring [3,4,5]. In recent years, the main methods for the determination of CTS were spectrophotometric methods [6,7,8] and HPLC methods [9,10,11]. Spectrophotometric methods have the advantages of simplicity and low cost, but they are not sensitive enough [12]. HPLC methods have the advantages of sensitivity and accuracy, but HPLC cannot directly determine CTS without hydrolysis. Thus far, 100% hydrolysis efficiency of CTS still cannot be achieved, which affects the accuracy of the determination of CTS and is the main imperfection in the use of HPLC to assay CTS. Therefore, it remains worthwhile to develop a highly sensitive, convenient, and rapid method for determining CTS.

Resonance Rayleigh scattering (RRS) is a special elastic scattering which is produced when the wavelength of Rayleigh scattering is close to the molecular absorption band [13,14]. It provides useful information concerning molecular structure, form, size, state of combination, charge distribution, and other factors [15]. RRS is a highly-sensitive analytical technique for the determination of certain inorganic [16,17,18] and organic substances [19,20,21].

**Figure 1:**
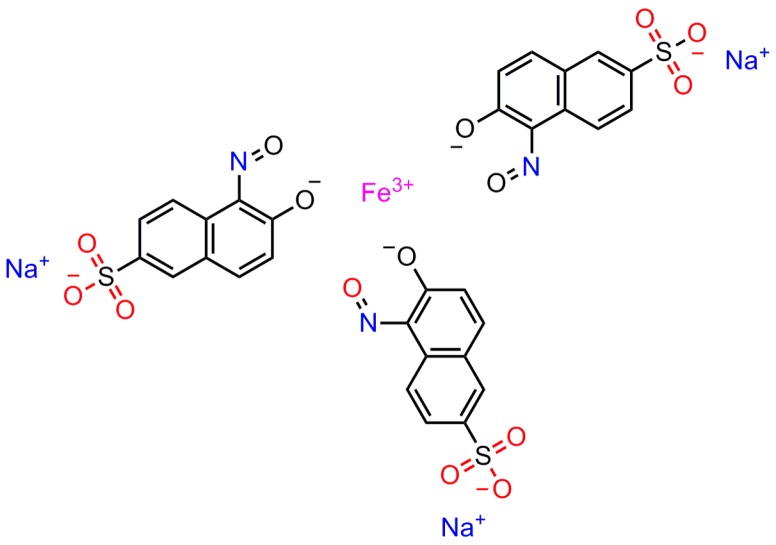
The structure of NGB.

Naphthol Green B (NGB) (Figure 1) is a complexometric indicator with three SO_3_^−^ groups and a naphthalene structure, leading to excellent water solubility and good stability. In this assay, it is the first time that NGB was proposed as a highly-sensitive probe for the determination of CTS. The experimental results have showed that both CTS and NGB produce very weak RRS signals. However, when the two agents react by virtue of electrostatic interaction to form an ion-association complex, the RRS intensity could be enhanced greatly. In this paper, the reaction principle, UV-VIS spectral, and RRS spectral characteristics, optimum reaction conditions, and analytical properties have been studied. It is worthwhile to mention in this context that the effect of the molecular weight of CTS was investigated. The experimental data analyzed through linear regression analysis has shown that there is no statistical significance on the molecular weight.

Therefore, CTS could be accurately quantified by this method even if the molecular weight of sample CTS is different from that of CTS standard. A highly sensitive method has been established and applied to the determination of complicated CTS capsules.

## 2. Results and Discussion

### 2.1. Mechanism

**Figure 2:**
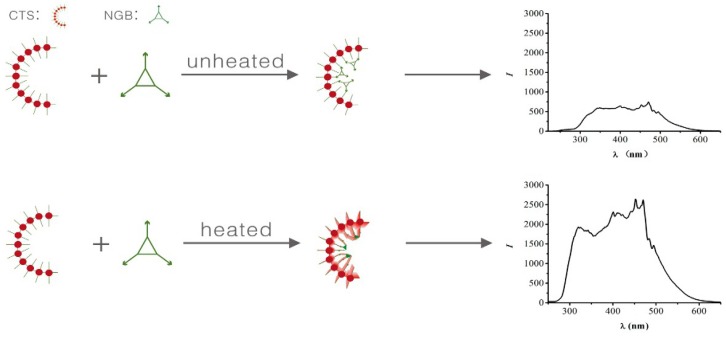
Schematic diagram of CTS-NGB system.

In this assay, the RRS method is used to test the change of CTS. The working principle of our sensing system is schematically represented in Figure 2. First, in an acidic solution, CTS becomes a positively-charged macromolecule as the –NH_2_ of CTS is protonated to –NH_3_^+^ [22]. NGB takes a negative charge on the surface [23].With positively-charged CTS in solution, so that it is very easy to form an ion-association complex, the scattering intensity is enhanced. In addition, when the CTS-NGB complex is placed in a 75 °C water bath for 3 min, the color of the CTS-NGB complex changes to yellow from green. When the solution is green, the CTS-NGB complex solution has greater molecular absorption above 600 nm. After heating and the solution turns yellow, the CTS-NGB complex solution has greater molecular absorption at 300–500 nm, almost overlapping with the scattering wavelength of the solution. Thus, the resonance between the absorption and the scattering is formed. As a result, the RRS intensity is greatly enhanced.

### 2.2. UV-VISAbsorption Spectra

**Figure 3:**
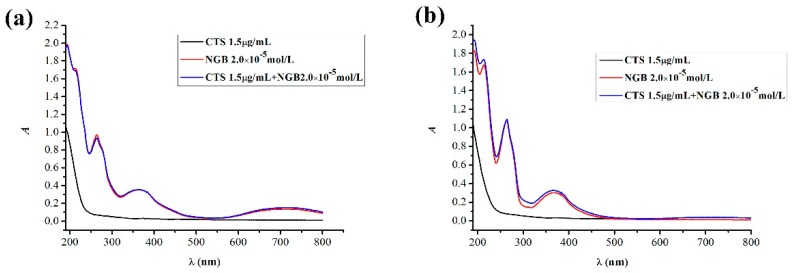
Comparison of the unheated and heated UV-VIS spectral characteristics of CTS-NGB system. (**a**) The UV-VIS absorption spectral characteristics for the unheated CTS-NGB system, and (**b**) the UV-VIS absorption spectral characteristics for the heated CTS-NGB system.

Figure 3 compares the unheated and heated UV-VIS spectral characteristics of the CTS-NGB system and shows that the unheated and heated CTS have almost the same UV-VIS spectral characteristics. In contrast to the CTS solution, the heated NGB solution has very different UV-VIS spectral characteristics from that of unheated NGB solution. The unheated NGB solution exhibits the maximum absorption peaks at λ_1_ = 264 nm, λ_2_ = 364 nm, and λ_3_ = 716 nm, whereas the heated NGB solution exhibits the maximum absorption peaks at λ_1_ = 264 nm and λ_2_ = 364 nm. Thus, the heated NGB solution undergoes a color change from green to yellow which is visible to the naked eyes. These results all confirmed that the spatial structure and chromophoric group of NGB might be changed in the process of heating. The UV-VIS spectral characteristics of CTS-NGB complex solution unheated and heated are similar to that of the pure NGB solution, but after heating, the absorbance of CTS- NGB complex solution is slightly higher than that of pure NGB solution.

### 2.3. RRS Spectra

**Figure 4:**
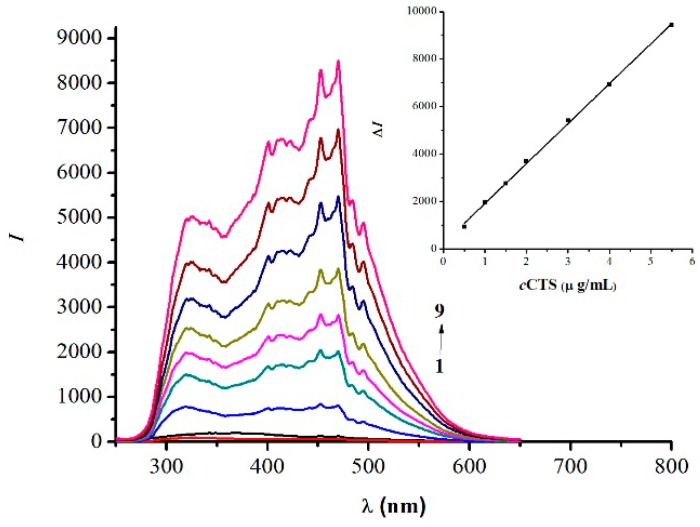
RRS spectra of the CTS-NGB dye systems.1. CTS 2.0 µg/mL; 2. NGB2.0 × 10^−5^ mol/L; 3–9. CTS (0.5, 1.0, 1.5, 2.0, 3.0, 4.0, and 5.0 µg/mL) –NGB (2.0 × 10^−5^ mol/L) complex.

Figure 4 depicts the RRS spectra of the CTS-NGB system and shows that the RRS intensities of CTS and NGB solution were individually weak under the measurement conditions. When CTS reacted with NGB to form an anion-association complex, the RRS was enhanced remarkably, and a new spectrum appeared. The maximum RRS peak was at λ = 470 nm, and the enhancement of RRS intensities was proportional to the concentration of CTS. Therefore, the new method of monitoring CTS could be established.

### 2.4. Optimum Experimental Conditions

In the experimental conditions optimization process, we chose a medium molecular weight chitosan as standard.

#### 2.4.1. Effects of Buffer Solution

**Figure 5:**
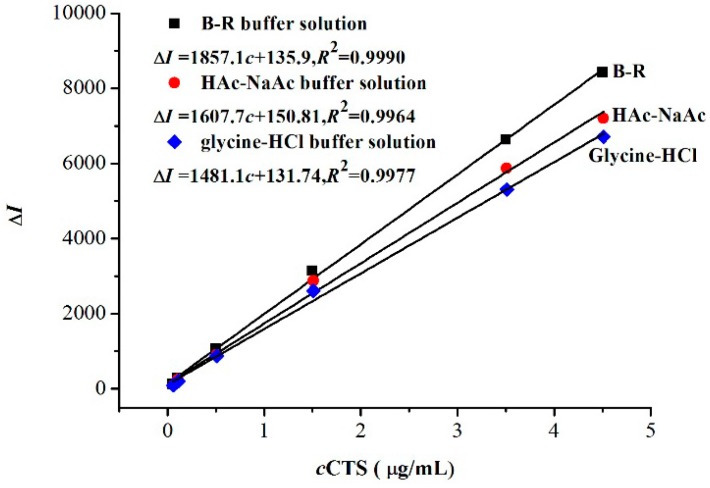
Effects of buffer solution. (■) B-R buffer solution; (●) HAc-NaAc buffer solution; (♦) Glycine-HCl buffer solution; The pH of three buffer solutions is 2.0; *t =* 75 °C.

Three buffer solutions, namely, HAc-NaAc buffer solution, glycine-HCl buffer solution, and B-R buffer solution, were used to investigate the influence of acidity on Δ*I* and the linear relationship of the standard curve in the CTS-NGB system (Figure 5). The results showed that B-R buffer solution was the most suitable reaction medium and it was, therefore, selected for further study.

#### 2.4.2. Effects of pH and Amount of B-R Buffer Solution

When B-R buffer solution was used as the reaction medium, the scattering intensity (Δ*I*) of CTS-NGB complex solution was the strongest in the pH range 1.9–2.5. When the pH value was outside this range, Δ*I* decreased. The pH 2.0 buffer solution was chosen as the reaction medium for the following experiments, and the most suitable amount of buffer solution was 1.5 mL for the reaction system.

#### 2.4.3. Effect of Concentration of Naphthol Green B

By increasing the amount of NGB in the experimental solution, the RRS intensity of this system was increased first and then decreased (the concentration of CTS was 5.5 μg/mL). The results indicated that the optimum concentration range was (1.0−3.0) × 10^−5^ mol/L. When the concentration of NGB in the solution was lower than 1.0 × 10^−5^ mol/L, the RRS intensity of the solution was decreased because of the CTS in the solution not completely reacted. When the concentration of NGB was higher than 3.0 × 10^−5^ mol/L, Δ*I* decreased. Δ*I* was the strongest at concentration of 2.0 × 10^−5^ mol/L, so this concentration was chosen as the optimum concentration for the reaction system.

#### 2.4.4. Effect of Reaction Temperature

**Figure 6:**
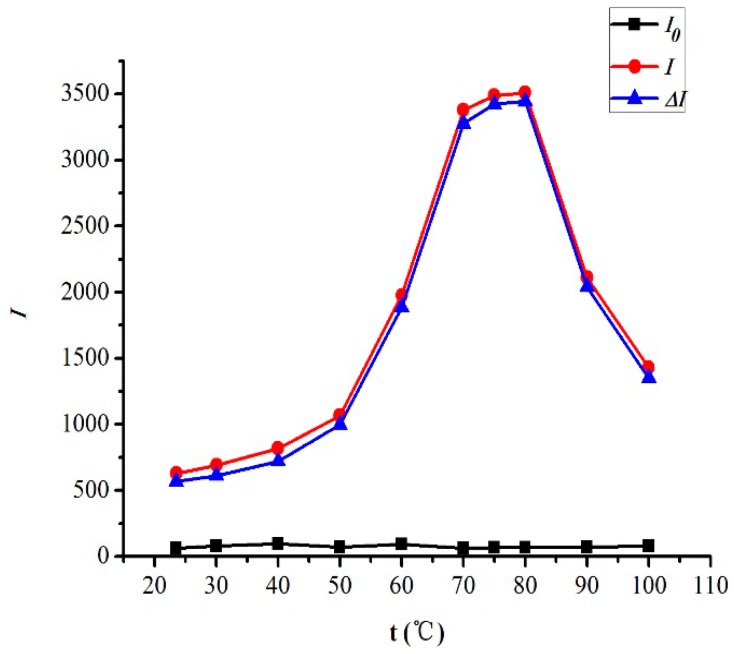
Effects of reaction temperature. CTS 2.0 µg/mL, NGB 2.0 × 10^−5^ mol/L, B-R pH = 2.0, 1.5 mL.

The effect of reaction temperature on the RRS intensity was examined. Figure 6 displays the results at room temperature (23.5 °C), 30 °C, 40 °C, 50 °C, 60 °C, 70 °C, 75 °C, 80 °C, 90 °C and 100 °C, which shows that temperatures had a great influence on the RRS intensity. When the temperature was 70–80 °C, the RRS intensity was stronger. However, when the temperature was higher than 80 °C, the RRS intensity decreased significantly. Therefore, 75 °C was selected as the optimum reaction temperature for the CTS-NGB system.

#### 2.4.5. Effects of Reaction Time and Stability

Under the optimum experimental conditions, the reaction time in a 75 °C water bath and stability at room temperature were studied. The CTS-NGB system reacted completely in 3 min and could remain stable for 4.0 h at room temperature. These results indicated that this system reacted quickly at 75 °C and exhibited good stability.

#### 2.4.6. Effect of Addition Sequence

Under the optimum experimental conditions, three addition sequences of reagents were tested: first, CTS-B-R buffer solution-NGB; second, CTS-NGB-B-R buffer solution; and third, NGB-B-R buffer solution-CTS. The experimental results of the three sets clearly showed that the RRS intensity of the third addition sequence was the highest. The following experiments all use NGB-B-R buffer solution-CTS as the addition sequence.

#### 2.4.7. Effect of Ionic Strength

**Figure 7:**
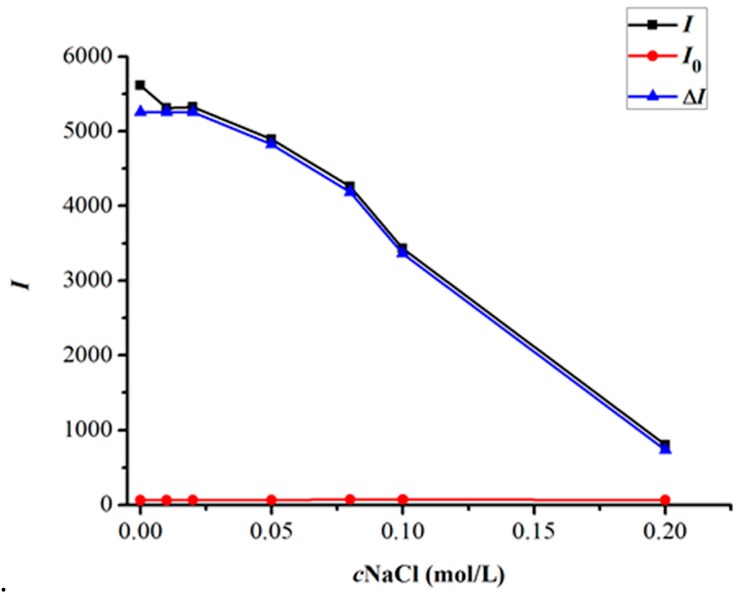
Effect of ionic strength on the RRS intensities of the CTS-NGB system. CTS 3.0 µg/mL, NGB 2.0 × 10^−5^ mol/L, B-R pH = 2.0 1.5 mL, *t* = 75 °C

The effect of ionic strength on the CTS-NGB system was tested by varying the concentration of NaCl. As shown in Figure 7, when the concentration of NaCl was controlled below 0.03 mol/L, the determination results of the CTS-NGB system were relatively stable. When the concentration of NaCl was increased, the RRS intensity decreased. The reason was that large amounts of Na^+^ cations and Cl^−^ anions in solution would be combined with CTS and the anion dye to form ion complexes, opposing the binding of CTS with the anion dye.

### 2.5. Analytical Application

#### 2.5.1. Calibration Curves

Under the optimum conditions, CTS of variant concentrations reacted with NGB to form ion-association complexes, and Δ*I* was measured after 3 min reaction in a 75 °C water bath. A calibration curve of Δ*I* against CTS concentration over a certain range was constructed: Δ*I* = 1860.5*c* + 86.125 (*c*, µg/mL).The correlation coefficient *R^2^* = 0.9999, and the linear range and the detection limit(DL) were 0.01–5.5 µg/mL and 8.87 ng/mL, respectively. This work and other methods for the determination of CTS are compared in Table 1, which shows that this method exhibits higher sensitivity and will be a valuable tool for the determination of CTS.

#### 2.5.2. Effect of the Molecular Weight of CTS

Three kinds of chitosan with different molecular weights (low, medium, high) were selected as standards, and a series of concentrations (0.1, 0.5, 1.5, 3.5, and 4.5 µg/mL) were prepared for each solution. The reagent blank was prepared at the same time, and all the solutions were determined under the optimum experimental conditions. A series of calibration curves of Δ*I* against the concentrations of CTS were constructed. Finally, the results were analyzed by linear regression analysis using the statistical product SPSS20.0 (IBM Company, Armonk, NY, USA) to determine the effect of the molecular weight of CTS. The results showed that *p* = 0.224 > 0.05, which suggested that the effect of molecular weight on the determination results has no statistical significance. Thus, the molecular weight does not interfere with the determination of CTS, even though there are significant differences in molecular weight between the CTS sample and the CTS standard.

#### 2.5.3. Effect of the Degree of Deacetylation

When the degree of deacetylation of chitosan is greater than 85%, its biological activity and solubility are better. Therefore, the degree of deacetylation of chitosan applied to the health food was more than 85%, generally.

Two kinds of chitosan with different deacetylation degree (the molecular weight of CTS is 60CPS and the degree of deacetylation are 85% and 90%) were selected as standards. Two calibration curves were constructed as in 2.5.2. The linear regression analysis results exhibited *p* > 0.05, which showed that the effects of different degree of deacetylation of chitosan on the determination results were not statistically significant.

#### 2.5.4. Effect of Foreign Substances

CTS capsule is composed of chitosan and a small amount of other auxiliary components. Under the optimum conditions, the effects of certain common foreign substances on the determination of CTS capsules were detected, and the results are given in Table 2, which shows that when the concentration of CTS was 3.0 µg/mL, some metal ions such as Fe^3+^, Ca^2+^, Mg^2+^, Cu^2+^, and Zn^2+^ were only tolerated in small amounts. Other additives, such as glucose, glycine, l-lysine, and l-tryptophan, even if there are large amounts, will not affect the accurate determination of chitosan, In order to eliminate the interference of metal ions, we chose tartaric acid and EDTA as masking agents. Firstly, we studied the effect of tartaric acid or EDTA on the dose. The results are shown in Table 3. In addition, we compared one set having tartaric acid or EDTA with another set not containing the two masking agents by *t*-test. The result of *t*-test was *p* > 0.05, which showed that the addition of tartaric acid or EDTA had no effect on the determination results.

For unknown samples, it is difficult to know what interfering substances are in the sample. However, we know that the presence of metal ions will increase the conductivity of the sample solution. Therefore, we added metal ions Fe^3+^, Ca^2+^, Mg^2+^, Cu^2+^, and Zn^2+^ (amount tolerated in Table 2), respectively, in the chitosan standard solution with a concentration of 3.0 µg/mL and detected the electrical conductivity. The results were 433.7 ± 25.54 µs/cm, 431.7 ± 16.56 µs/cm, 444.7 ± 5.507 µs/cm, 427.7 ± 6.351 µs/cm, and 535.3 ± 9.451 µs/cm.

We also detected the electrical conductivity of two CTS sample solutions (Olevy chitosan capsules, AiDeLan chitosan capsules), the results were 490.0 ± 5.196 µs/cm and 490.7 ± 8.083 µs/cm, which showed that there might be some interfering substances in the samples. However, when the concentration of EDTA in the sample solutions was 0.001 mol/L, the conductivity of the solutions was smaller than 200 µs/cm. At this point, the relative error of the measurement results can be controlled less than 5%.

#### 2.5.5. Precision and Recovery

The method was applied for the determination of CTS in health products, specifically Olevy and AiDeLan CTS capsules. The CTS capsules were weighed accurately to obtain 0.4 g and dissolved in a 100-mL volumetric flask with 0.5 mol/L HAc for 36 h. Then, 10.00 mL was obtained after high speed centrifugation as a sample stock solution. Next, 2.50 mL stock solution was added to a 100-mL volumetric flask, diluted to the mark with high-purity water and thoroughly mixed to produce a working solution.

The following operations were similar to the general procedure with 0.5 mL working solution used as a determination sample (with EDTA added as a masking agent). The corresponding results were calculated according to the calibration graphs of CTS, and the results are listed in Table 4. Moreover, the recoveries of CTS in this method were investigated, and the results are listed in Table 5. The recoveries of the Olevy capsule and AiDeLan capsule were 103.2%–104.6% and 102.7%–103.5%, respectively, and the relative standard deviations were 0.71% and 0.42%, respectively. Thus, this method exhibited good recovery and reproducibility.

## 3. Experimental

### 3.1. Materials and Reagents

Three types of chitosan (CTS, low molecular weight: ≤200 mPa.S, medium molecular weight: 200–400 mPa.S, high molecular weight: 400–1000 mPa.S; 1% in 1% acetic acid, 20 °C; Sigma, St. Louis, MO, USA). The stock solution of 400.0 µg/mL chitosan was prepared by mixing a suitable chitosan and 0.5 mol/L HAc solution, and a working solution of 10.0 µg/mL chitosan was prepared for use in the experiment. A working solution of Naphthol Green B (NGB, 2.0 × 10^−4^ mol/L, Tokyo Chemical Industry Co. Ltd., Tokyo, Japan) was prepared and kept at 4°C. Britton-Robinson (B-R) buffer solutions with different pH values were prepared by combining the mixed acid (consisting of 2.71 mL of H_3_PO_4_, 2.36 mL of HAC and 2.47 g of H_3_BO_3_)/L with 0.2 mol/L NaOH in different proportions, and the pH values were adjusted using a pH meter. Olevy chitosan capsules (Weihai South Gulf Biological Co. Ltd., Weihai, China) and AiDeLan chitosan capsules (Shanghai TongJi Biological Co. Ltd., Shanghai, China) were used. All reagents were of analytical grade without further purification, and high-purity water was used throughout.

A Hitachi F-2500 spectrofluorophotometer (Hitachi Ltd., Tokyo, Japan) was used for recording RRS spectra and measuring RRS intensity. A UV-3010 spectrophotometer (Hitachi Ltd., Tokyo, Japan) was used to record the absorption spectra and measure the absorbance. A PHS-3C pH meter (Shanghai Scientific Instruments Company, Shanghai, China) was used to measure the pH values of the solutions, and a CP124C electronic analytical balance (Ohaus Instrument Co. Ltd., Shanghai, China) was used in this experiment.

### 3.2. Procedures

Appropriate amounts of NGB, B-R buffer solution, and CTS were added to a 10 mL cuvette. Then, each cuvette was diluted with water to a final volume of 10.0 mL. The solution was mixed and set in a 75 °C thermostat water bath for 3 min, followed by rapid cooling to room temperature. The RRS spectra were recorded by scanning synchronously with the same excitation and emission wavelengths and measuring RRS intensity by time scan pattern. The slit (λ_ex_ = λ_em_) was 5 nm/5 nm, and the PMT was 400 V. The RRS intensity *I* was measured for the reaction product and *I*_0_ for the reagent blank at the maximum RRS wavelength, Δ*I* = *I−I*_0_.

## 4. Conclusions

In conclusion, a novel and innovative methodology was developed to quantify CTS and successfully applied to the determination of CTS samples. The main advantages of this assay are that it is simple, sensitive, accurate, and rapid. Vitally, the molecular weight and the deacetylation degree of CTS exhibit no interference with its accurate quantification. The detection limit (DL) of the method is good, and the RSD of the method is better. In contrast to HPLC methods, this method does not require sophisticated pretreatment processes.

## Figures and Tables

**Table 1:** Comparison of sensitivities for determination of CTS between present method and some other methods.

Methods	Linear Range	Detection Limits	Investigated the Effect of the Molecular Weight of CTS	References
UV-VIS spectra	10–80 µg/mL	_-_	N	[6]
UV-VIS spectra	4.55–30.30 mg/L	1.41 mg/mL	Y	[24]
Cathodic Stripping Voltammetry	5.0 × 10^−7^–1.7×10^−5^ g/mL	5.0 × 10^−7^ g/mL	N	[25]
RRS	0.042–3.0 µg/mL	1.2 µg/mL	N	[26]
SOS	0.016–3.0µg/mL	4.9 µg/mL	N	[27]
FDS	0.005–3.0 µg/mL	1.6 µg/mL	N	[27]
RRS	0.10–20.0 µg/mL	29 ng/mL	N	[27]
HPLC	(2–20 mg/mL) Glucosamine	_-_	N	[11]
RRS	0.01–5.5 µg/mL	8.87 ng/mL	Y	This paper

RRS: Resonance Rayleigh scattering, SOS: second-order scattering, FDS: frequency doubling scattering, HPLC: High-performance liquid chromatographic, N: No, Y: Yes.

**Table 2:** Effects of foreign substances (*c*_CTS_: 3.0 µg/mL).

Foreign Substance	Amount Tolerated (µg/mL)	RE (%)	Foreign Substance	Amount Tolerated (µg/mL)	RE (%)
Glucose	9600.00	−3.59	Fe^3+^	0.30	−4.76
β-Cyclodextrin	120.00	3.33	Mg^2+^	0.40	−3.67
Soluble starch	100.00	−5.51	Ca^2+^	0.40	4.64
VitaminC	85.00	−4.39	K^+^	10.00	3.33
Citricacid	37.50	−5.05	Na^+^	5.00	−3.38
Glycine	1600.00	4.69	Cu^2+^	0.12	3.84
l-lysine	900.00	−5.38	Zn^2+^	0.80	−5.07
l-tryptophan	250.00	3.42	NH_4_^+^	441.85	−5.02
l-leucine	135.00	−4.01			

**Table 3:** The results of anti-interference experiment (*c*_CTS_: 3.0 µg/mL).

Cation	The Concentration of Cation (µg/mL)	Masking Agent	The Concentration of Masking Agent	RE (%)
Fe^3+^	3.0	EDTA	0.001 mol/L	−4.22
Mg^2+^	2.0	Tartaric acid	0.08%	4.78
Ca^2+^	3.0	Tartaric acid	0.08%	2.88
Cu^2+^	3.0	Tartaric acid	0.02%	1.31
Zn^2+^	3.0	EDTA	0.001 mol/L	−3.04

**Table 4:** Results of the determination of CTS capsules.

Sample	Olevy (mg/g)	AiDeLan (mg/g)
1	920.2	871.5
2	938.5	871.1
3	930.1	878.4
4	927.2	876.7
5	922.4	877.2
6	925.6	871.8
Average (mg/g)	927.3	874.5
RSD (%)	0.70	0.38

**Table 5:** The results of recoveries.

Sample	Found Value (µg/mL)	Added(µg/mL)	Found Value (µg/mL) (*n*=6)	Recoveries (%)	RSD (%)
Olevy	0.4658	0.4000	0.8830	104.3	0.71
		1.000	1.512	104.6	
		1.500	2.014	103.2	
AiDeLan	0.4498	0.4000	0.8605	102.7	0.42
		1.000	1.485	103.5	
		1.500	2.001	103.4	
